# Recent Advances in Microrobots Powered by Multi-Physics Field for Biomedical and Environmental Applications

**DOI:** 10.3390/mi15040492

**Published:** 2024-04-02

**Authors:** Xiangyu Teng, Zezheng Qiao, Shuxuan Yu, Yujie Liu, Xinyu Lou, Huanbin Zhang, Zhixing Ge, Wenguang Yang

**Affiliations:** 1 School of Electromechanical and Automotive Engineering, Yantai University, Yantai 264005, China; 15166828596@163.com (X.T.); qiaojingbiao6656@163.com (Z.Q.); ysx17753172676@163.com (S.Y.); 15106690585@163.com (Y.L.); 17860387799@163.com (X.L.); zhb13031740719@163.com (H.Z.); 2 State Key Laboratory of Robotics, Shenyang Institute of Automation, Chinese Academy of Sciences, Shenyang 110016, China; gzx-01@nus.edu.sg

**Keywords:** microrobots, soft actuator, motion forms, biomedical applications

## Abstract

Microrobots powered by multi-physics fields are becoming a hotspot for micro–nano manufacturing. Due to the small size of microrobots, they can easily enter small spaces that are difficult for ordinary robots to reach and perform a variety of special tasks. This gives microrobots a broad application prospect in many fields. This paper describes the materials, structures, and driving principles of microrobots in detail and analyzes the advantages and limitations of their driving methods in depth. In addition, the paper discusses the detailed categorization of the action forms of microrobots and explores their diversified motion modes and their applicable scenarios. Finally, the article highlights the wide range of applications of microrobots in the fields of biomedicine and environmental protection, emphasizing their great potential for solving real-world problems and advancing scientific progress.

## 1. Introduction

The use of robots in modern society has become widespread and diverse, ranging from industrial robots to service robots. Industrial robots are now essential to the production process, as they increase productivity, ensure processing accuracy, and reduce the burden on human laborers [1,2]. Service robots are also becoming increasingly important in everyday life [3]. However, there are situations where they may need to be more suitable, such as within the human body, where their size makes it impossible for them to operate effectively. As technology advances, we witness an emerging branch of the robotics field microrobots.

Microrobots possess the unique advantage of being able to access confined spaces that are challenging for traditional robots to reach, allowing them to carry out specialized tasks with precision and efficiency [4,5,6,7,8,9,10,11,12]. Their small size makes it difficult to realize their drive through batteries and motors. To overcome these problems, a widely investigated solution is to utilize the power of physics fields, which involves the manipulation of physics fields to achieve precise control and functional enhancement of microrobots. Their exceptional agility and flexibility enable them to navigate and maneuver freely within small and intricate environments, making them well suited for applications in a wide range of industries. The advantages of physics field drive are mainly reflected in its flexible energy conversion and drive mechanism. These microrobots are capable of responding to various types of fields, such as magnetic fields [13,14], acoustic fields [15], light fields [16], and chemical fields [17,18,19]. As a result of this multifaceted responsiveness, these microrobots are capable of executing an extensive repertoire of motion patterns, including linear motion [20], circular motion [21], and spiral motion [22]. This dynamic athleticism allows them to easily travel through complex and confined spaces, demonstrating their potential for deployment in a wide range of applications.

The microrobots can be categorized as powered by single-physics fields and powered by multi-physics fields. The motion of microrobots powered by single-physics fields is relatively simple, stable, and easy to control. However, due to the limitation of single-physics field drive, microrobots are relatively single-functional and cannot perform complex tasks and movement patterns. On the other hand, microrobots powered by multi-physics fields are able to realize more complex and diversified movement modes, enabling them to perform more complicated tasks. Through the synergy of multi-physics fields, microrobots can realize more efficient collaboration and perform better in dealing with complex environments and tasks.

Machine learning technology plays a significant role in solving the challenge [23] in the development of microrobots. It gives microrobots greater decision-making power and solves many challenges. Through deep learning models, microrobots can automatically identify and track targets, enhancing collaboration and monitoring capabilities [24]. The use of machine learning algorithms to achieve the autonomous navigation of microrobots, including obstacle avoidance, path planning, and other functions, improves their movement ability and the ability to adapt to complex environments [25,26].

**Figure 1:**
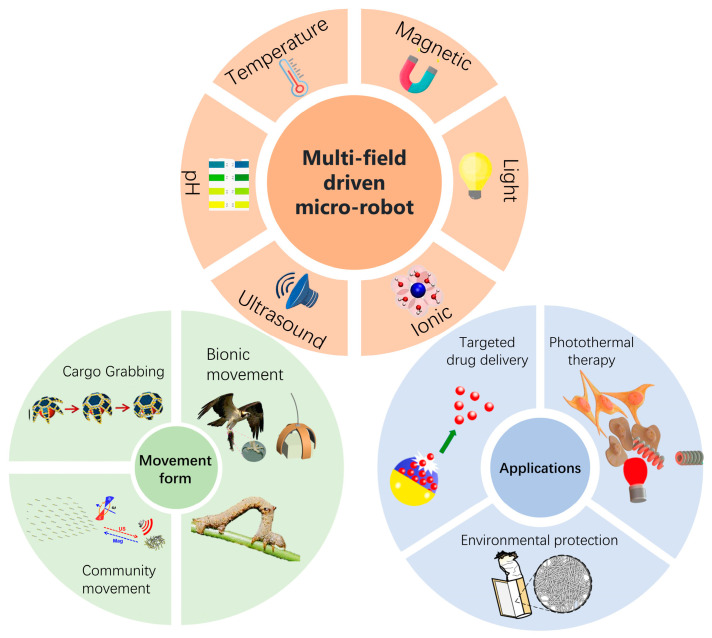
Overview of microrobots powered by multi-physics fields, including drive fields, forms of microrobot motion, and applications of microrobots. Cargo grabbing reproduced from reference [30] with permission from the *Journal of Micro-Bio Robotics*. Community movement reproduced from reference [31] with permission from *Nano Letters*. Bionic movement reproduced from reference [32] with permission from *Nature Communications*. Targeted drug delivery reproduced from reference [33] with permission from the *Chemical Engineering Journal*. Photothermal therapy reproduced from reference [34] with permission from *Small*. Environmental protection reproduced from reference [35] with permission from *Small Methods*.

In this article, we systematize microrobots powered by multi-physics fields from different perspectives (Figure 1). We comprehensively discussed microrobots powered by dual-physics fields and three-physics fields. The materials, structures, and response principles of microrobots are described in detail through specific cases. The article then delves into various forms of motion of the microrobots, including grasping, transporting, and releasing goods, group motion, and simulating biological motion. Finally, we focus on the prospects of microrobots powered by multi-physics fields in medical [27,28] and environmental protection [29] fields, analyzing their potential applications in precision drug delivery, photothermal therapy, and pollutant removal.

## 2. Classification of Microrobots Powered by Multi-Physics Fields

### 2.1. Microrobots Powered by Dual-Physics Fields

#### 2.1.1. Microrobots Powered by Magnetic and pH

Microrobots powered by magnetics and pH utilize the properties of magnetic and pH-sensitive materials to achieve precise motion control and localization. pH-sensitive materials can change their morphology or properties according to the surrounding environment, thus realizing the control of the microrobot. Microrobots utilize materials with magnetic properties, often incorporating ferromagnetic or paramagnetic elements. Therefore, the microrobots will deform and move under the action of the magnetic field. In this section, we describe the materials, structures, and response principles of magnetic and pH-responsive microrobots.

**Figure 2:**
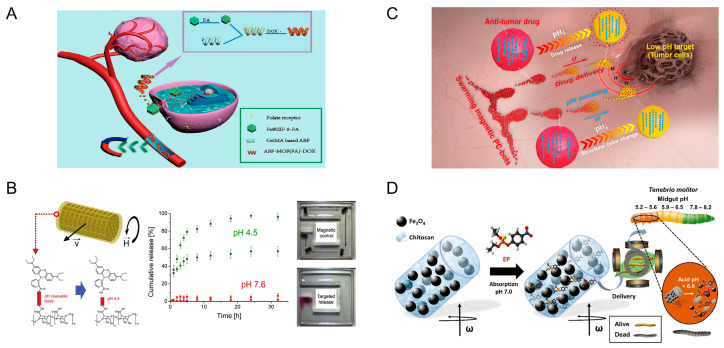
Microrobots powered by magnetic and pH. (**A**) Magnetic microrobots with folate targeting for drug delivery. Reproduced from reference [37] with permission from *Cyborg and Bionic Systems*. (**B**) Coating of magnetically controlled microdevices with functionalized alginate-based hydrogels. Reproduced from reference [39] with permission from *Materials and Design*. (**C**) Swarming magnetic photonic-crystal microrobots. Reproduced from reference [40] with permission from *InfoMat*. (**D**) Hydrogel chitosan magnetic microrobots encapsulating ethyl parathion (EP)-CHI@ Fe_3_O_4_ are used to efficiently kill mealworm larvae. Reproduced from reference [43] with permission from *Small*.

An example of microrobots that respond to pH changes are those based on hydrogel structures. Li et al. fabricated a double-layered soft microrobot. This microrobot consists of 2-hydroxyethyl methacrylate (PHEMA) pH-responsive hydrogel and poly (ethylene glycol) diacrylate (PEGDA) with iron oxide particles (Fe_3_O_4_). The PHEMA layer is a pH-sensitive hydrogel, while PEGDA containing iron oxide particles can be excited by magnetic fields. In the pH range of 7.0–9.0, the robot is spherical and able to encapsulate drugs inside. At lower pH, the layer loses water and shrinks, and the robot undergoes unfolding motion to release the drug particles. The magnetic excitation of the robot is achieved by the electromagnetic actuation (EMA) system. The controllability of magnetic excitation is proved by experiments, which show that the robot can pass through the H-shaped chamber under the control of the EMA system [36]. Drug release through hydrogel deformation is a viable option. Ye and his team designed magnetic microrobots with folate targeting for drug delivery. In this study, folic acid (FA) was introduced into the microrobot to promote endocytosis, which is key for drug entry into cells. These microrobots are prepared from biodegradable gelatin methacryloyl (GelMA) and a magnetic metal–organic framework (MOF). The porous structure of the MOF and the polymerized GelMA hydrogel network are used to load enough FA and the anticancer drug doxorubicin (DOX), respectively. With the control of a rotating magnetic field (20 mT, 2 Hz), these microrobots were able to gather around the lesion site, and the targeting effect of FA combined with the magnetic navigation effect significantly improved the anticancer efficiency of these microrobots (Figure 2A) [37]. Hydrogels have suitable biocompatibility and controllability, making them a promising pH-responsive material.

The changing of pH also has an effect on chemical bonding, and this principle can be utilized to enable the control of microrobots. Jin et al. proposed a microrobot for the treatment of hemangiomas. This microrobot consists of a mixture of pH-responsive self-healing hydrogel matrix and magnetic nanoparticles. The microrobots were guided by an external magnetic field to bring them together. Experiments have shown that the optimal range for magnetic swarm control is from 2 to 5 Hz. At low pH, the interaction between the carboxyl and amide groups triggers the formation of hydrogen bonds between the microrobots, which combine the microrobots into a large, spherical structure [38]. Targeted drug delivery can improve the relevance and efficacy of treatments while reducing unwanted side effects. Bernasconi et al. describe magnetically controlled microdevices that enable targeted drug delivery. The cylindrical scaffold of the microdevice deposits four metal layers: an initial copper layer, a CoNiP layer with magnetic properties, a NiP layer, and an Au layer that provides biocompatibility. The metal layer is wrapped with a hydrogel layer, and the drug is linked to the hydrogel by ester or amide bonds. The bond cleavage can be increased under acidic pH to release the drug. By applying an external magnetic field to the hydrogel microrobots, controlled movement within the tiny channel can be achieved (Figure 2B) [39]. The ability to detect environmental pH while targeting drug delivery is a huge breakthrough. Yu et al. designed swarming magnetic photonic-crystal microrobots (PC-bots). The magnetic PC-bots consist of pH-responsive hydrogel microspheres and Fe_3_O_4_ nanoparticles encapsulated inside. They can self-organize into large swarms by controlling an external rotating magnetic field. The team studied the movement speed of the microrobot when the magnetic induction intensity was 0–20 mT. The formation of the swarms originates from the lateral hydrodynamic attractive and repulsive forces in the x-y plane. The hydrogels of the microrobots undergo protonation and deprotonation when the ambient pH changes; their volume and lattice constants change accordingly, and the color of light diffraction changes as a result. The PC robot is in a solvated state at pH 7.4. Doxorubicin (DOX) molecules are linked to the carboxylate sites on the hydrogel scaffolds by electrostatic forces. At pH 6.5 and 5.0, the carboxylate sites of the robot are protonated. The electrostatic force interaction is weakened, and the DOX drug molecule is released (Figure 2C) [40].

Some materials can be dissolved in specific pH environments, and utilizing these materials to create microrobots can enable the release of goods in specific pH environments. Microrobots and metal–organic frameworks (MOFs) have been recognized as promising vehicles for drug delivery applications. Terzopoulou et al. designed MOF-based small-scale robots (MOFBOTs) for biomedical applications. The MOF used in the study not only enabled superior loading of chemotherapeutic drugs and controlled release via pH-responsive degradation but also controlled movement of enzymatically biodegradable gelatin-based helical microrobots under magnetic fields (8 mT) [41]. Similarly structured magnetic and pH-responsive microrobots were also designed by Wang et al. The difference is that they used zinc-based MOF [42]. In addition to the medical field, these microrobots also have applications in the field of environmental protection. Pests pose a serious threat to agriculture, ecosystem, and human health. Maria-Hormigos and her team have designed magnetic hydrogel microrobots to achieve targeted killing of pests. The outer layer of the microrobots is made of chitosan hydrogel. The interior is encapsulated with ethyl parathion (EP) and Fe_3_O_4_ particles. The microrobots are powered by a transversely rotating magnetic field (5 mT, 5 Hz) that allows them to be evenly distributed in the food of the pest larvae. The chitosan hydrogel swells and dissolves at an acidic pH (<6.0). This allows the microrobots to break down and release EP in the acidic environment of the pest gut (Figure 2D) [43].

Microrobots powered by magnetics and pH move through changes in the magnetic field or pH and have low energy dependence. Localization of tumor cells and improved drug release efficiency can be achieved by changes in pH. However, pH may fluctuate abnormally in the human body, which leads to the possibility of releasing drugs at the wrong location.

#### 2.1.2. Microrobots Powered by Magnetic and Temperature

Microrobots powered by magnetic and temperature showcase a dynamic fusion of external control and thermal adaptability. In response to changes in temperature, they produce changes in shape or volume to achieve the corresponding maneuvers. In this section, we describe the structure and response principles of magnetic and temperature-responsive microrobots.

**Figure 3:**
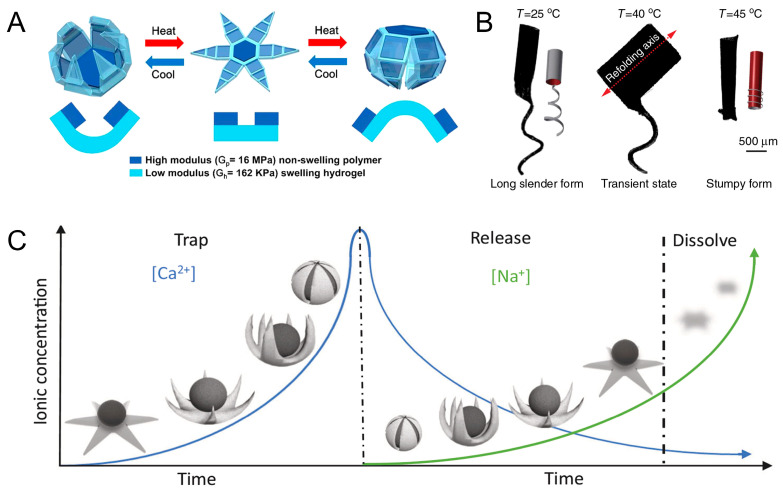
Microrobots powered by magnetic and temperature and microrobots powered by magnetic and ionic. (**A**) Self-folding thermo-magnetically responsive soft microgrippers. Reproduced from reference [44] with permission from *ACS Applied Materials & Interfaces*. (**B**) Soft micromachines with programmable motility and morphology. Reproduced from reference [45] with permission from *Nature Communications*. (**C**) Ionic shape-morphing microrobotic end-effectors. Reproduced from reference [48] with permission from *Nature Communications*.

Combining a flexible layer with a rigid layer is a common design solution for this type of robot. Breger et al. designed a two-layer microgripper. The temperature-responsive layer of the microgripper is a pNIPAM-AAc soft hydrogel. Iron oxide (Fe_2_O_3_) nanoparticles are embedded in a porous hydrogel layer, allowing the microgripper to respond and be guided remotely using a magnetic field. The material of the rigid layer is polypropylene fumarate, which can increase the rigidity of the robot so that the microrobot can be used to excise cellular tissues. At temperatures below 36 °C, the hydrogel layer absorbs water and swells. The microgripper expands as it absorbs water and then contracts and closes in the opposite direction (Figure 3A) [44]. Precise control of the shape of a microrobot is a difficult part of current research, so the study of shape control is of great significance. In 2016, Huang et al. designed customizable shaped 3D bionic micro-swimmers, microrobots containing non-expandable poly (ethylene glycol) diacrylate (PEGDA). It responds thermally by a N-isopropylacrylamide (NIPAAm) hydrogel layer. Magnetic nanoparticles (MNPs) were added to the hydrogel layer to induce the micro-swimmer motion. In particular, the selective alignment of MNP nanoparticles can produce different folding axes. The thermal response of the micro-swimmer to structural changes can be altered by programming the folding axes. When heated, the micro-machine can reversibly change its form between a long, slender form and a stumpy form (Figure 3B). Magnetic propulsion of the microrobot can be realized by generating magnetic driving force through an eight-coil electromagnetic manipulation system. The maximum uniform magnetic field generated by the system is 40 mT, and the maximum magnetic field gradient is 1 T/m [45]. The team also designed hydrogel soft microrobots with self-folding capabilities. The microrobots use similar structures and principles as the 3D bionic micro-swimmers [46]. Yoshida et al. designed spiral micro-swimmers that mimic the flagella of microorganisms. The micro-swimmer consists of a stimulus-responsive gel and a non-responsive gel. The spiral micro-swimmer propels itself by rotational motion excited by a rotating magnetic field (10 mT and 5 Hz). The expansion and contraction behavior of the bilayer spiral gel depends on the cross-sectional pattern of the stimulus-responsive hydrogel. The stimulus-responsive hydrogel undergoes reversible contraction or expansion due to thermal stimulation, which in turn changes the pitch and speed of movement of the micro-swimmer [47].

#### 2.1.3. Microrobots Powered by Magnetic and Ionic

Ions, such as calcium and sodium ions, can cause changes in the hydrogel network. When combined with a magnetic field, it can be used to control microrobots. Inspired by sea star predation on conch, Zheng et al. designed ionic shape-morphing microrobotic end-effectors (ISMEs) with ion response to shape change. ISMEs utilize an ALG-based ion-responsive hydrogel to respond to ionic gradients. At the same time, the MNPs are encapsulated in the ISMEs to achieve controlled movement under a magnetic field (3–20 mT). These microstructures are immersed in a CaCl_2_ solution, which crosslinks with Ca^2+^, causing the structure to shrink. Subsequently, they are placed in a sodium citrate solution where Ca^2+^ is displaced, and the gel structure is allowed to expand. This endowed it with the ability to exhibit self-curling and self-folding at different ionic concentrations. Therefore, ISME is able to implement grab and release functions (Figure 3C) [48]. The cargo and the microrobot are actually two separate entities. Another way is to integrate the microrobot and the cargo into one. Spiral microcapsules were prepared, and the core of the microcapsules was a calcium alginate hydrogel network. The exterior is a biocompatible alginate–chitosan–alginate (ACA) shell decorated with Fe_3_O_4_ nanoparticles. When it encounters sodium citrate solution, ion exchange between Ca^2+^ and Na^+^ occurs, and the calcium alginate hydrogel core liquefies and swells. The expansion force generated by the liquefaction swelling causes the ACA membrane to stretch and thin. Because the ACA membrane at the end of the spiral microcapsules is relatively thin, the encapsulated drug is released from the end of the spiral structure. When the magnetic field strength is 8 mT, the microcapsules can be rotated under the control of a six-degrees-of-freedom electromagnetic system at different frequencies and can follow a planned path of spiral motion [49].

Microrobots powered by magnetics and ionics combine magnetic field guidance with sensitivity to changes in ion concentration, providing the ability to maneuver precisely in complex environments. However, these robots are relatively difficult to operate and control. The complexity of chemical changes in the human body leads to the microrobot’s ionic switches being triggered in the wrong place, which can lead to unexpected problems.

#### 2.1.4. Microrobots Powered by Light and Magnetic Fields

**Figure 4:**
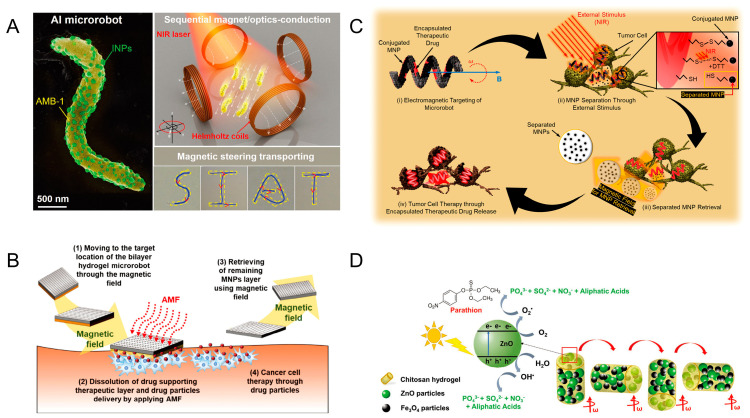
Microrobots powered by light and magnetic fields. (**A**) Sequential magneto-actuated and optics-triggered biomicrorobots. Reproduced from reference [50] with permission from *Advanced Functional Materials*. (**B**) Soft magnetic microrobots for photoactive pollutant removal. Reproduced from reference [54] with permission from *Small Methods*. (**C**) Bilayer hydrogel microrobots capable of retrieving MNPs after drug delivery. Reproduced from reference [56] with permission from *Advanced Healthcare Materials*. (**D**) Magnetically actuated drug delivery helical microrobots with magnetic nanoparticle retrieval ability. Reproduced from reference [57] with permission from *ACS Applied Materials & Interfaces*.

Microrobots powered by light and magnetic fields integrate materials that respond to specific wavelengths of light. When illuminated, these materials undergo photochemical or photothermal reactions. Most of them realize the position movement by a magnetic field and realize the corresponding function by a light response. Bacteria exist in nature in a variety of forms, and these bacteria provide biological templates for humans to build microrobots. Xing et al. used magnetospirillum magneticum (AMB-1) to create magnetically driven light-responsive microrobots. The researchers chemically conjugated AMB-1 to light-triggered indocyanine green nanoparticles (INPs) via a Michael addition reaction. It is capable of being directed autonomously to the tumor site by the effects of internal hypoxia and externally applied magnetic fields (200 mT). The INPs can convert light energy into heat energy under the irradiation of a near-infrared laser to kill tumor cells (Figure 4A) [50]. Akolpoglu et al. designed bacterial biohybrids using *E. coli* as a template. MNP nanoparticles and nanoliposomes (NLs) encapsulated with the anticancer drug doxorubicin (DOX) were assembled with *E. coli* through non-covalent interactions. They applied different external magnetic field strengths (5, 10, and 20 mT) to bacterial biohybrids to investigate whether the field strength has an impact on motility. The lipid bilayer structure of NL transforms from an ordered gel phase to a disordered liquid crystal phase under near-infrared light irradiation, at which point the drug is released [51].

The use of photocatalytic reactions allows for the propulsion of microrobots and the release of cargo, and the combination of light and magnetism allows for the control of microrobots. Villa et al. report on microrobots for the removal of yeast from beer. These microrobots used a star-shaped photosensitive material (BiVO_4_) decorated with Fe_3_O_4_ nanoparticles. They can be magnetically driven without fuel. The microrobots rotate when a horizontally rotating magnetic field is applied, and they produce directional motion under a vertically rotating magnetic field. Under external light conditions, the microrobots undergo a photocatalytic reaction and generate propulsion. Light also triggers the ability of the microrobots to capture yeast cells from the environment. Powered by the combination of light and magnetic fields, the microrobot removes almost 100% of the remaining yeast cells [52]. Light can break chemical bonds, and light-induced drug delivery is common in such microrobots. Bozuyuk et al. proposed a magnetically powered, double-helical micro-swimmer. Chitosan, with excellent biocompatibility and biodegradability, was used as the microrobots’ body. There are magnetic nanoparticles in the mesh structure of the body of the micro-swimmer. Drug molecules are chemically bound to photocleavable linker molecules on the body. The micro-swimmer allows controlled movement under a rotating magnetic field of 10 mT. Under bright light, the photocleavable linker splits into two parts, resulting in 60 percent of the drug being released from the micro-swimmer within five minutes [53]. Degradation of pollutants can also be achieved through photocatalytic reactions. Maria-Hormigos et al. designed CHI@ Fe_3_O_4_-ZnO microrobots for pollutant degradation. Chitosan (CHI) was used as the main body of the microrobots with Fe_3_O_4_ nanoparticles and ZnO nanoparticles. Fe_3_O_4_ nanoparticles were used to actuate the microrobots in response to an external magnetic field, while ZnO nanoparticles were used for the degradation of pollutants. The CHI hydrogel adsorbs the surrounding pollutants, and the ZnO acts as a photocatalyst to decompose the POP pollutant parathion by UV light irradiation. The microrobot moves rapidly under a higher-frequency magnetic field. The solution is rapidly mixed by the microrobot, which increases the rate of photocatalytic degradation of pollutants (Figure 4B) [54].

Magnetic nanoparticles are often harmful to the human body and need to be removed from the body after the corresponding operation in the human body. In 2019, Kim et al. designed microrobots with recoverable MNP. The hydrogel microrobots reach the lesion site through the EMA system. The gelatin was then decomposed by near-infrared light irradiation, leaving only MNP and PLGA-DOX drug particles in the target area. The MNP is then recovered by the EMA system [55]. A year later, the team presented a two-layer hydrogel robot capable of recovering magnetic nanoparticles after drug release. The miniature hydrogel robot consists of a layer of MNPs and a therapeutic layer. The therapeutic layer hydrogel contains PLGA-DOX drug particles. After the microrobot reaches the target site by magnetic navigation, an alternating magnetic field (AMF) is applied to the microrobot to increase its temperature. The high temperature causes the hydrogel of the therapeutic layer to dissolve and release the drug. The MNP layer was subsequently recovered by the magnetic field. The microrobot could freely locomote in the x-y plane by the 20 mT magnetic field and the 2 mT m^−1^ gradient magnetic field, while it could move in the x-z plane by the magnetic field of 45 mT and the gradient magnetic field of 8 mT m^−1^ (Figure 4C) [56]. Lee et al. designed a drug transportation robot capable of MNP recovery. The microrobot is based on biocompatible PEGDA with an internal encapsulation of the anticancer drug DOX. MNP is bound to the surface of the microrobot through disulfide bonds. The microrobot is controlled by an integrated system of eight-coil EMA and NIR. The microrobot is moved to the tumor cell area by electromagnetic manipulation. By applying NIR light stimulation to cause the disulfide bonds to break, the MNPs were separated from the body of the microrobots and recovered by a magnetic field (25 mT) (Figure 4D) [57].

Microrobots powered by light and magnetic fields can be operated with a high degree of control precision and flexibility. The application of magnetic fields endows them with the ability to navigate stably in complex environments. However, magnetic materials may have negative side effects on the human body and need to be removed from the body. The drug loaded on the microrobot may not be fully released due to insufficient light intensity.

#### 2.1.5. Microrobots Powered by Light and pH

Microrobots powered by both light and pH offer a unique blend of stimuli-responsive capabilities, merging optics and chemical reactivity. The use of light and pH to control microrobots can improve the adaptability and expand the functionality of microrobots. Power et al. developed a microrobot for cancer targeting and drug delivery. The microrobot consists of cylindrical pH-responsive polymer and claws fabricated from photoresponsive hydrogels. The assembly of the charged cationic polyelectrolyte with the uncharged hydrophilic polymer forms a highly stable layer. These layers are sensitive to low-pH environments due to the electrostatic repulsion generated between them. When photoresponsive hydrogels are immersed in water, water molecules fill the entire porous material. The hydrogel undergoes isomerization after light exposure. The hydrogel becomes more hydrophobic, resulting in the exclusion of water molecules [58]. When two types of physics fields are used to propel a microrobot, the microrobot can better adapt to its environment.

This kind of microrobot is able to reduce dependence on external energy sources and save energy costs. They can accurately localize target areas and perform delicate operations. In the medical field, they can be used for tasks such as localization and therapeutic drug release. Challenges include optimizing response times and ensuring stability in varying conditions. Ongoing research aims to overcome these hurdles, paving the way for advancements in microrobotics with dual-stimuli propulsion.

#### 2.1.6. Microrobots Powered by Ultrasound and Magnetic Fields

The acoustic and magnetic fields used to drive the microrobots are below safety standards, so they are safe for the human body. By utilizing the compound effect of ultrasound and magnetic fields, two forces can be utilized simultaneously to drive a microrobot. The flexibility and versatility of the microrobots’ movement is increased. Both ultrasonic waves and magnetic fields have a strong ability to propagate and act in different environments. This enables the microrobots to adapt to various scenarios and task requirements.

**Figure 5:**
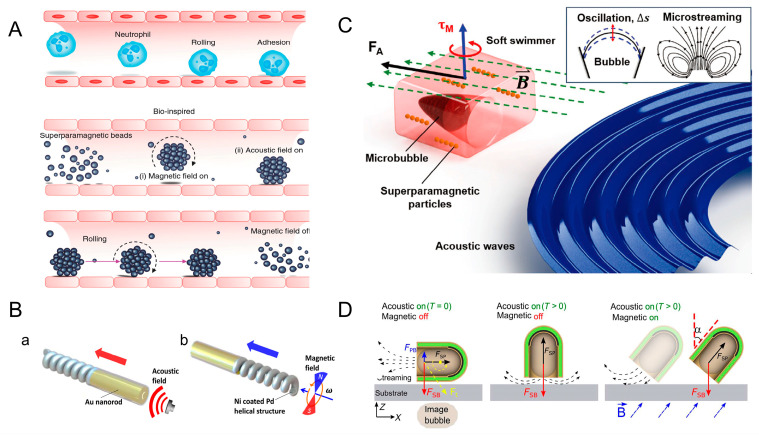
Microrobots powered by ultrasound and magnetic fields. (**A**) Bio-inspired rolling motion by introducing superparamagnetic particles in magnetic and acoustic fields. Reproduced from reference [59] with permission from *Nature Communications*. (**B**) Magneto-acoustic hybrid nanomotor. (a) The Au nanorod powered by acoustic field. (b) Ni coated Pd helical structure powered by magnetic field. Reproduced from reference [31] with permission from *Nano Letters*. (**C**) Artificial acousto-magnetic soft micro-swimmers. Reproduced from reference [60] with permission from *Advanced Materials Technologies*. (**D**) Magnetic- and ultrasound-driven micro-swimmer. Reproduced from reference [61] with permission from *Science Advances*.

Inspired by neutrophils, in 2017, Ahmed et al. simulated the process using superparamagnetic particles. In the presence of a rotating magnetic field (10–20 mT), dipole–dipole interaction occurs between superparamagnetic particles. This interaction causes the particles to cluster together to form spherical structures. Subsequently, the micro-group will move toward the sound pressure node under the action of the acoustic field. By combining these two physics fields, the rolling of the microrobot along the pipe boundary can be realized (Figure 5A) [59]. Most of the microrobots driven by ultrasound have a special shape so that they can be driven by the sound pressure gradient generated by ultrasound. Li et al. designed a magneto-acoustic hybrid fuel-free nanomotor, which contains concave Au nanorod segments and a magnetic helical structure. The concave Au nanorod segments are necessary for ultrasonic actuation. Ultrasonic waves acting on the concave end generate a propulsive force in the direction of the red arrow. This transducer generates a vertical acoustic standing wave at a resonant frequency of about 2.66 MHz. (Figure 5(Ba)). The nanomotor moves in the direction of the blue arrow under the action of the rotating magnetic field (Figure 5(Bb)) [31]. RBC cells in the human body can also be used as carriers in response to ultrasound waves. Wu et al. designed RBC motors using RBC cells. Iron oxide nanoparticles were loaded into the RBC cells, where they were asymmetrically distributed. Since red cells have asymmetry, RBC motors can be driven by the sound pressure gradient generated by ultrasound. The RBC motor can be driven in different media at an ultrasound voltage of 3V and a frequency of 2.93 MHz. Directional control of RBC motors can be achieved by applying an external magnetic field. Experiments have shown that RBC motors only rotate in situ when only a magnetic field is applied. Under the combined effect of ultrasound and magnetic field, RBC motors exhibit controlled motion [20].

Ahmed et al. have designed a micro-swimmer that contains a microcavity inside. This soft micro-swimmer contains one or more microcavities located in the center of its main body and carries superparamagnetic particles within its polymer matrix. Air bubbles are captured inside the microcavity. When the micro-swimmer is exposed to an acoustic field, the bubbles oscillate and generate fluid propulsion. The frequencies used in the experiment for propulsion were in a range between 20 and 22 kHz. The sequential arrangement of magnetic particles can change the direction of motion of the micro-swimmer in response to an external magnetic field (10–15 mT) (Figure 5C) [60]. Ren et al. reported a micro-swimmer that controls the direction by the combined action of a magnetic field and acoustic field. A layer of magnetic nickel is deposited in the micro-swimmer, and its capsule structure contains air bubbles. When an acoustic field is applied to the micro-swimmer, the bubbles inside vibrate. This produces a primary Bjerknes force FPB, a streaming propulsive force FSP, and a secondary Bjerknes force FSB, which is negligibly smaller than the FSP and is oriented toward a rigid interface. The direction of the FSP can be changed by changing the pointing of the micro-swimmer through an external magnetic field, and the direction of the combined force of the FSB and FSP is the direction of advancement of the micro-swimmer. In a magnetic field with a fixed direction (α = 45°), the micro-swimmer reached its fastest speed at a frequency of 1.33 MHz (Figure 5D) [61].

#### 2.1.7. Microrobots Powered by Light and Ultrasonic Fields

By rationally designing the structure of a microrobot, it can be made to move in opposite directions under the control of different physics fields. Tang et al. designed hybrid light/acoustic-powered microbowl motors, which are composed of gold (Au) and titanium dioxide (TiO_2_). Sharp-edged oscillations in the acoustic field (2.66 MHz, 5 V) generate a second-order acoustic flow, which causes the microbowl to advance to its outer side. In hydrogen peroxide, UV light activates photochemical reactions on the peptide dioxide surface. The generation of hydrogen ions on the TiO_2_ surface and their depletion on the Au surface leads to an asymmetric distribution of ions around the microbowl, which drives the micromotor [62]. Light can control not only the movement of microrobots but also the release of drugs. Garcia-Gradilla et al. reported ultrasound-driven nanowire motors, which achieve drug release by near-infrared light irradiation. The nanowire motors were composed of four segments, including solid Au, Ni, and Au segments, as well as a porous Au one. The solid end of the nanowire motors has a concave geometry. An asymmetric pressure gradient is generated by ultrasonic waves, and the propulsive force of the nanowire motors is generated as a result (using ultrasound power and frequency of 6 V and 2.01 MHz, respectively). Plasma resonance and photothermal effects occur on the localized surface of nanoporous gold. The conversion of absorbed light energy into heat can induce local structural changes in the anionic polymer and lead to effective drug release [63].

### 2.2. Microrobots Powered by Three-Physics Fields

**Figure 6:**
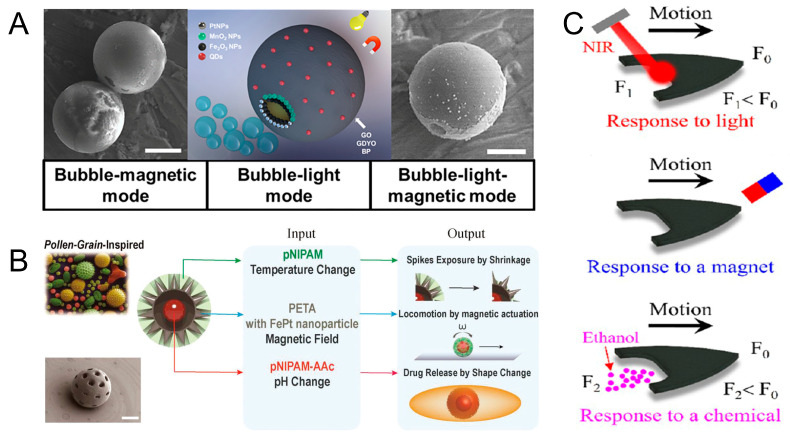
Microrobots powered by three-physics fields. (**A**) Janus micromotors with built-in multiengines for bubble, magnetic, and light-driven propulsion. Reproduced from reference [65] with permission from *Chemistry of Materials*. (**B**) Grain-inspired robots that respond to magnetic field, temperature, and pH. Reproduced from reference [66] with permission from *Advanced Materials*. (**C**) Multistimuli-responsive hydroplaning superhydrophobic microrobots. Reproduced from reference [67] with permission from *ACS Nano*.

Microrobots that can respond to the three-physics field have excellent adaptability and flexibility [17,18] and can realize complex multi-dimensional movements [64]. Yuan et al. designed a micromotor wrapped with gold-sputtered polystyrene microspheres through two-dimensional nanomaterials. Pt or MnO_2_ nanoparticles are assembled on the surface of the micromotor to act as a bubble (catalytic) engine. Fe_2_O_3_ nanoparticles are used as a magnetic engine. Quantum dots (QDs) are used as light-driven engines. Pt or MnO_2_ in hydrogen peroxide solution can be used as a catalyst to generate oxygen, which drives the micromotor to move. By irradiation with UV light, the electrons released by the QDs are trapped in the catalytic layer, resulting in a negative net charge on the metal side, which reacts with the oxygen and protons present in the medium to produce additional H_2_O_2_. In addition, the micromotor can be guided by an external magnetic field for motion to occur (Figure 6A) [65]. Inspired by the structure of pollen, Lee et al. designed a 3D-printed multifunctional microrobot, which consists of a three-layer hydrogel structure: iron platinum (FePt) nanoparticle-embedded pentaerythritol triacrylate (PETA), poly N-isopropylacrylamide (pNIPAM), and poly N-isopropylacrylamide acrylic acid (pNIPAM-AAc). The outermost layer of pNIPAM absorbs water and swells when the temperature falls below the low critical solution temperature (LCST). At this time, the microrobot is spherical and can roll and steer in response to a magnetic field. When the temperature is below LCST, the hydrogel layer loses water and shrinks, leaking spikes from the shell. With the help of leaking spikes, the microrobots can be held in place. The microrobot can achieve pH-responsive cargo release when the pH changes (Figure 6B) [66]. The spherical structure allows the microrobot to move in any direction. This design allows the microrobot to traverse complex environments and obstacles with greater flexibility. The spherical structure design provides a higher degree of freedom, allowing the microrobot to perform tasks in more innovative and varied ways, as well as providing greater scope for future technological developments.

Wang et al. designed multistimuli-responsive hydroplaning superhydrophobic microrobots. These microrobots are controlled by light, magnetic, and chemical fields. The microrobots were composed of Polydimethylsiloxane (PDMS) and graphene and nanoparticles (Fe_3_O_4_). Upon irradiation of the end of the microrobots with near-infrared light, the graphene converts the light energy into heat energy, which leads to an increase in the temperature of the water. According to the Marangoni effect, the surface tension of water decreases with increasing temperature. Microrobots are propelled because of the imbalance of forces on them. In the presence of a magnetic field, the microrobot can achieve controlled motion. When ethanol molecules come into contact with water, the surface tension of the liquid decreases, which propels the microrobots (Figure 6C) [67]. Alapan et al. fabricated micro-swimmers by combining erythrocytes and *E. coli*. Erythrocytes were used as cargo carriers, which were internally loaded with anticancer drugs and iron oxide nanoparticles. The *E. coli* and erythrocytes were linked together by biotin–avidin–biotin. The micro-swimmer is driven by the rotation of the flagellum of *E. coli* and oriented by an external magnetic field (20 mT). Since *E. coli* proliferates rapidly and uncontrollably, irradiation with near-infrared light is used to inactivate the *E. coli* after reaching the target location. The release of anticancer drugs is accelerated in lower pH environments. This is due to the swelling of RBCs osmotically and their eventual hemolysis [68].

Through a combination of physics fields such as magnetic, pH, light, and ultrasound, these robots can achieve more sophisticated and flexible control methods and adapt to more diverse application environments. These microrobots are capable of performing highly precise operations such as precision drug delivery. However, the cooperative control of multi-physics fields increases the complexity of the system and requires more advanced design and regulation techniques.

## 3. Action form of Microrobot

### 3.1. Grabbing, Transporting, and Releasing Cargo

**Figure 7:**
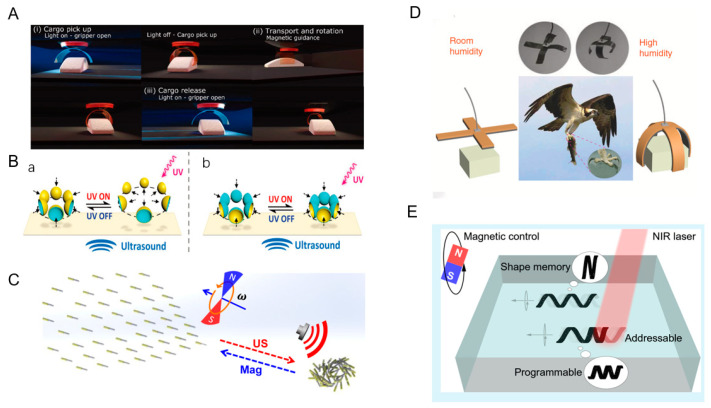
Multiple forms of motion for microrobots. (**A**) Gripping, transporting, and releasing of goods by microrobots. Reproduced from reference [69] with permission from *Advanced Optical Materials*. (**B**) Collective motion of microrobots powered by light and ultrasonic fields. (a) Schematic illustration of dispersion behavior of resulting swarm of microbowl. (b) Compaction of resulting swarm of microbowls upon UV illumination. Reproduced from reference [62] with permission from *Advanced Functional Materials*. (**C**) Collective motion of microrobots powered by magnetic and ultrasonic fields. Reproduced from reference [31] with permission from *Nano Letters*. (**D**) Simulated eagle claw movement. Reproduced from reference [32] with permission from *Nature Communications*. (**E**) Variable pitch movement. Reproduced from reference [73] with permission from *ACS Applied Materials & Interfaces*.

Microrobots need to overcome size constraints, surface adhesion forces, and inertia to achieve accurate grasping and precise object transportation. Cunha et al. reported a soft robotic gripper that is synergistically controlled by light and magnetism. It enables gripping, transporting, rotating, and releasing of cargo. The soft robotic gripper consists of a combination of light-responsive and magnetic-responsive layers. The soft robotic gripper opens the gripper by light when it is over the cargo. When the light is turned off, the soft robotic gripper automatically closes and grabs the goods. The magnetic field is used to guide the gripper to have translational and rotational degrees of freedom in motion. After reaching the target position, the light is applied again, the soft robotic gripper opens automatically, and the goods are released (Figure 7A) [69]. Adding a rigid layer to a soft gripper can increase the gripping capacity of the soft gripper. Ongaro et al. designed magnetically and temperature-responsive soft untethered grippers. It enables the gripping transport and release of materials in porcine tissues. The soft, untethered gripper was first transported over the target tissue by magnetic navigation. The soft, untethered gripper turns into a sphere during the gripping task. The cargo is then guided by a magnetic field to complete the transportation. After reaching the release area, the temperature is lowered, and the gripper opens and releases the cargo. After the above operations, the gripper can be removed from the organism by the magnetic field [30].

Grab and release can also be realized through the attraction between particles in the acoustic field. Ren et al. designed acoustically powered micro-swimmers for single-particle manipulation. The micro-swimmers have two different modes of operation: push and pull. The push mode is realized at low sound pressure. In this mode, the micro-swimmer moves toward the center of the target particle, and the target can be propelled and transported. The particle is released by moving the micro-swimmer away from the particle. The pull mode is realized at higher acoustic pressure. The attraction between the particle and the micro-swimmer becomes larger, and the micro-swimmer can attract the particle when it is close to the particle. Both can move together to complete the transportation task. After reaching the target position, the target particle can be released by further tilting the micro-swimmer to increase the repulsive flow between them [61].

### 3.2. Collective Movement

Collective movement is common in nature, and animals often adopt this behavior in order to better survive and reproduce. Researchers have designed micromotors to mimic this natural phenomenon. A common way to achieve collective movement is by aggregating toward acoustic pressure nodes. Tang et al. fabricated hybrid light/acoustic-powered microbowl motors with two different structures. The microbowl motors migrate along the sound pressure gradient to the low-pressure region of the sound field, so they cluster together. Under the irradiation of UV light, the TiO_2_-Au microbowls move away from each other, and the Au-TiO_2_ moves closer (Figure 7B) [62]. Collective movement can also be realized by the magneto-acoustic hybrid nanomotor designed by Li et al. The nanomotors all exhibit helical motion in the presence of a rotating magnetic field. Switching off the external magnetic field and applying an acoustic field causes the hybrid motors to rapidly converge toward the acoustic field nodes, and this convergence is caused by the acoustic pressure gradient. Turning off the acoustic field and applying a magnetic field causes the group to rapidly disperse and move in the same direction (Figure 7C) [31]. Aggregation of groups can also be realized by the mutual fusion of bubbles induced by an acoustic field. A small bubble is assembled at the end of the tubular micromotor, which can drive the micromotor under the action of ultrasonic waves. When many micromotors are close to each other, the small bubbles interact with each other to produce large, vibrating bubbles, and many micromotors can thus form a dandelion-like group and move together [70].

### 3.3. Biomimetic Microrobots

Over the course of billions of years of evolution, nature has created a variety of different forms of living things. Microrobots can simulate their functions by being driven by physics fields [71]. Eagle claws usually have a strong grip to hold prey or other objects tightly. Dong et al. designed GO/PPy bilayer actuators that can respond to temperature, humidity, and light to simulate an eagle’s claw. The gripper was structured horizontally to simulate the spreading of the eagle’s claws in indoor humidity and curved to simulate the eagle’s claws grasping the prey in high humidity (Figure 7D). In addition, the team simulated a tendril climber plant, which can change shape in response to changes in humidity. It can wrap around and grab the flower axis according to the geometry of the flower [32]. An inchworm is an insect larva that moves forward by curling and stretching its body. Zhang et al. designed soft actuators consisting of a low-density polyethylene (LDPE) layer and a graphene oxide (GO) layer to simulate the crawling of the inchworm. The robot was curled in the initial state and stretched under near-infrared light. When the light is turned off, the robot resumes its curled state. By repeating the above process, the robot can move forward slowly [72]. Drawing on the characteristics and behaviors of natural organisms can provide new possibilities for applications in a variety of physics fields, such as bionic robots that can play an important role in medical care, rescue, and detection. Pitch can be understood as the distance the helix advances per revolution. Zhao et al. presented a magnetic spiral microrobot based on shape memory polymers. The robot exhibits controlled motion under a rotating magnetic field. The photothermal effect can change the length, diameter, and chirality of the microrobots. Their diameter increases with the decrease in the number of turns (Figure 7E) [73].

## 4. Applications

Microrobots powered by multi-physics fields have a wide range of applications in the biomedical and environmental protection fields. Microrobots can go inside the human body and perform precise drug delivery, surgical operations, or disease diagnosis through positioning and control techniques, which has great potential in cancer treatment and vascular disease treatment. Microrobots can also be used for environmental cleaning and treatment. They can be used to clean the oceans of trash and pollutants, helping to protect marine ecosystems.

### 4.1. Biomedical Application

#### 4.1.1. Targeted Drug Delivery

**Figure 8:**
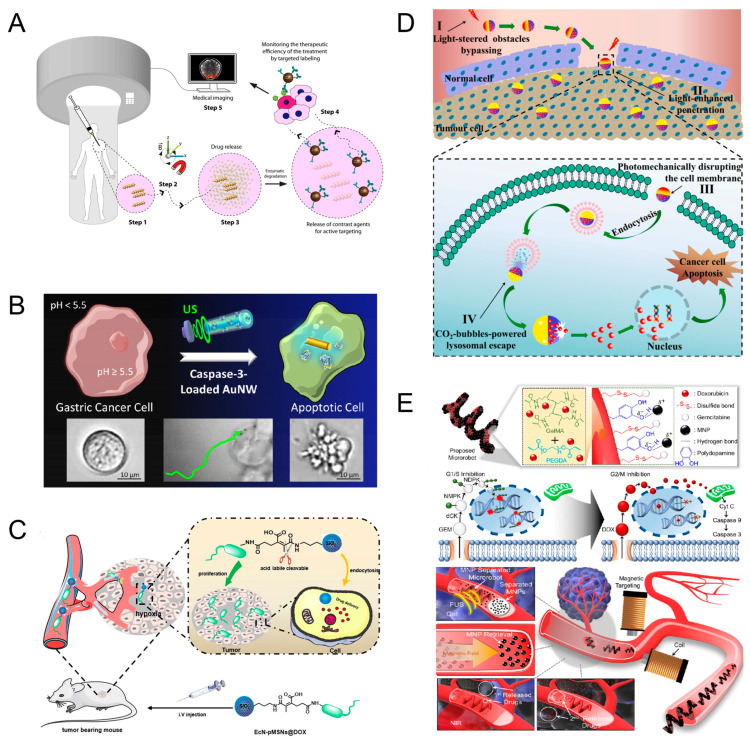
Targeted drug delivery. (**A**) Therapeutic procedures with microrobots. Reproduced from reference [78] with permission from *ACS Nano*. (**B**) Driving microrobots for drug release by ultrasound and pH. Reproduced from reference [79] with permission from *ACS Nano*. (**C**) Targeted drug delivery using *E. coli*. Reproduced from reference [80] with permission from *Colloids and Surfaces B: Biointerfaces*. (**D**) Light/gas cascade-propelled Janus micromotors that actively overcome sequential and multi-staged biological barriers for precise drug delivery. Reproduced from reference [33] with permission from *Chemical Engineering Journal*. (**E**) Microrobots with sequential dual-drug release capability. Reproduced from reference [81] with permission from *ACS Applied Materials & Interfaces*.

Microrobots powered by multi-physics fields could be used for precise drug delivery [74,75,76,77]. Targeted drug delivery can reduce the impact on healthy tissue while increasing the effectiveness of the treatment. The use of chemical changes to achieve tumor localization is an important approach to cancer treatment. In many cancers, tumor cells use matrix metalloproteinase-2 (MMP-2) enzymes to escape from the surrounding stroma; thus, local concentrations of MMP-2 are elevated. Ceylan et al. designed targeted drug delivery robots that can respond to pathological concentrations of MMP-2. The miniature robot has a double helix structure with the ability to swim under a rotating magnetic field. The micro-swimmer is injected near the tumor and is remotely controlled by an external magnetic field for precise navigation and localization of the tumor. Due to the elevated concentration of MMP-2 around the tumor, this promotes rapid swelling of the hydrogel network, which facilitates drug release. Antibody-modified magnetic contrast agent diffuses into the periphery to mark untreated tissue sites (Figure 8A) [78]. In addition to this, tumor cells can be localized using the acidic environment surrounding the tumor. Gastric adenocarcinoma is a malignant tumor within the stomach, which is characterized by an acidic extracellular space and a neutral intracellular cytoplasm. Based on this particular environmental condition, Esteban-Fernández de Ávila et al. designed gold nanowire (AuNW) motors with a pH-responsive polymer coating powered by ultrasound. The nanomotors were loaded with caspase-3 (CASP-3), which is a promising therapeutic protein that catalyzes the specific cleavage of key cellular proteins. In the acidic intragastric environment, the nanomotor protects the enzyme from inactivation before entering the cytoplasm. However, upon entry into the cell and exposure to higher intracellular pH, the polymer coating dissolves, which releases the active CASP-3 enzyme directly into the cytoplasm, leading to rapid apoptosis. Although this research is still in its infancy, in vitro experiments have shown that ultrasound-driven nanomotors may serve as an effective drug delivery method (Figure 8B) [79]. To address poor drug penetration, Wang et al. constructed a bacterially driven robot using *E. coli*. The *E. coli* and drug-loaded silica nanoparticles were linked together by pH-sensitive groups. *E. coli* recognizes signaling molecules in the hypoxic region of the tumor and aggregates and grows around the tumor cells. After the microrobots reach the perimeter of the tumor cells, the low-pH environment causes the pH-sensitive motifs to break, and the drug-loaded silica nanoparticles are released. This microrobot was experimentally demonstrated to have superior anti-tumor effects (Figure 8C) [80].

Light/gas cascade-propelled Janus micromotors designed by Zhou et al. can cross biological barriers for active drug release. The spherical structure of the micromotor is a calcium carbonate microsphere loaded with drugs, and a layer of gold is loaded on the surface of the microsphere. Under near-infrared light irradiation, thermophilic force is generated due to thermal gradient to propel the micromotor to move. The photothermal effect produced by light can make the micromotor penetrate the cell membrane. After entering the cell, the micromotor is endocytosed by lysosomes. The acidic environment in the lysosomes causes calcium carbonate to break down and release carbon dioxide gas, and with the help of gas propulsion, tiny motors can break through the lysosomes and release the drug. In the future, this technology may be a reliable option for selective tumor cell therapy (Figure 8D) [33]. To overcome treatment with only one drug, Lee et al. designed microrobots that can sequentially release dual drugs. Gemcitabine (GEM) is bound to the surface of the microrobots via disulfide bonds, and doxorubicin (DOX) is encapsulated inside the microrobots as the secondary drug release. In addition, MNP was attached to the surface of the microrobots for magnetic actuation (Figure 8E). The therapeutic principle of the drug is shown in (Figure 8E). During the treatment, the microrobot is guided around the tumor cells by a magnetic field. The heat generated by near-infrared light and glutathione surrounding the cancer cells can break disulfide bonds and release GEM. Over time, the microrobots slowly break down and release DOX (Figure 8E) [81]. Microrobots as a carrier for targeted drug delivery is an emerging technology, which has the advantages of precise drug delivery, reduction in systemic side effects, and enhancement of local therapeutic effects. However, the requirements for biocompatibility and stability are high, and there are still some challenges in clinical application.

#### 4.1.2. Photothermal Therapy

Photothermal therapy for tumor treatment can be realized by coating the surface of a microrobot with a layer that converts light energy into heat energy [82,83]. Microrobots used for photothermal therapy are often powered by magnetic fields and achieve therapeutic purposes through light response [84]. De la Asunción-Nadal et al. reported on the magnetic spiral microrobots (MoSBOTs). The surface of the microrobots is coated with molybdenum disulfide, which absorbs various wavelengths of light and converts them into heat. The microrobots can be guided by a magnetic field and swarm around the cancer cells, and by irradiating them with near-infrared light, they can generate localized high temperatures to destroy the tumor cells. Although this treatment is still in its infancy, it has great potential for destroying cancer cells [34]. A nanoscale liquid metal robot is described by Wang et al. The microrobot has a rod-like structure. It consists of a liquid gallium core and a solid gallium oxide shell. When exposed to an ultrasonic field, the acoustic radiation force generated in the suspension plane can propel them to move autonomously. Liquid metal nanorobots can actively seek out cancer cells and drill into them by drilling holes in them. As the reaction removed the gallium oxide layer due to the acidity inside the cancer cells, the nanorobots changed from rods to droplets. These transformed nanomachines can fuse together inside the cell and photothermally kill the cancer cells when exposed to near-infrared light [85].

The advantages of photothermal therapy include non-invasive, localized treatment, high controllability, low impact on surrounding tissues, and the ability to avoid the side effects of traditional treatments. However, photothermal therapy is only applicable to superficial tumors, and photosensitizers in the patient’s body may cause phototoxic reactions. Therefore, although photothermal therapy has obvious advantages in some cases, its limitations need to be further overcome and optimized.

### 4.2. Environmental Protection

Microrobots powered by multi-physics fields have great potential for environmental protection, including hormonal pollutants [86], microplastic [87,88], antibiotics [89], and other pollutants. They can be precisely controlled and localized using the action of multi-physics fields to achieve more efficient, precise, and sustainable environmental protection.

**Figure 9:**
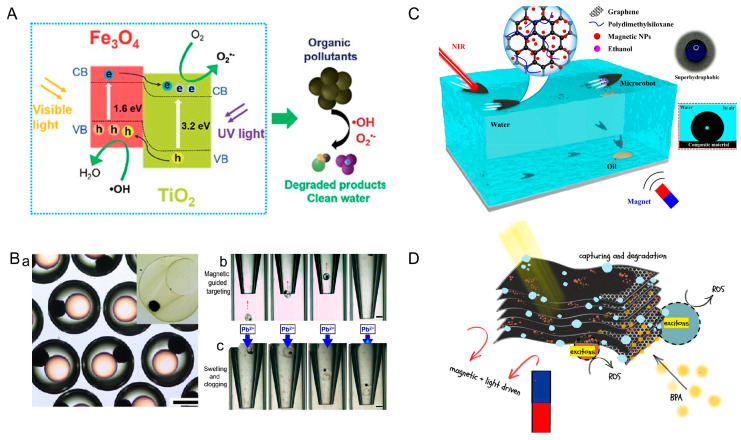
Applications of microrobots in environmental protection. (**A**) On-the-fly catalytic degradation of organic pollutants using magneto-photoresponsive bacteria-templated micro-cleaners. Reproduced from reference [90] with permission from the *Journal of Materials Chemistry A.* (**B**) Micro-actuators for Pb^2+^ leakage prevention. (a) The size distributions of the double emulsions. (b) Magnetic-guided targeting process of the microactuator. (c) Clogging of the microtube by Pb^2+^ responsive swelling of microactuator. Reproduced from reference [91] with permission from *ACS Applied Materials & Interfaces*. (**C**) Microrobot that enables oil spill recovery. Reproduced from reference [67] with permission from *ACS Nano*. (**D**) Microrobot for BPA removal. Reproduced from reference [35] with permission from *Small Methods*.

Freshwater is an important natural resource for human survival; however, non-naturally degradable organic pollutants in freshwater are accumulating year by year, and human health is under great threat. It is crucial to design materials that can catalyze these pollutants. Mushtaq et al. designed micro-helical robots composed of peptide dioxide and Fe_3_O_4_. When the microrobots are exposed to light, electron migration occurs, and a series of redox reactions take place. Oxygen and water are converted into highly reactive oxygen species such as superoxide and hydroxyl radicals, and organic pollutants are directly degraded by these radicals into harmless products (Figure 9A) [90]. Long-term consumption of plumbum-containing water can cause anemia, neurological damage, and other health problems. Liu et al. designed micro-actuators that can respond quickly to changes in plumbum ion concentration. The micro-actuator uses a hydrogel that is sensitive to Pb^2+^ concentration and has an eccentric core added to it for magnetic actuation (Figure 9(Ba)). The micro-actuator was guided to the Pb^2+^ leak port by a magnetic field (Figure 9(Bb)). An increase in the concentration of Pb^2+^ around the leakage port causes the hydrogel to swell, thereby plugging the leakage port. In addition to Pb^2+^, the micro-actuator can be made to respond to different ions by changing the hydrogel material. This study provides a viable platform for managing water pollution caused by different ions [91].

Oil spills from oil tankers occur from time to time, and the spills of crude oil cause great damage to the marine ecology. Multistimuli-responsive hydroplaning superhydrophobic microrobots provide a new idea for recovering oil pollution. The microrobots can move under the control of light, magnetic, and chemical fields. Through the coupling of multi-physics fields, the microrobots can achieve complex motions. This microrobot has suitable adaptability to complex environments and has a promising application in the field of oil recovery (Figure 9C) [67]. The recovery of marine oil pollution not only protects the marine ecosystem and safeguards human health but also helps to promote the sustainable development of the marine industry. Through effective oil pollution recovery efforts, threats to marine biodiversity can be reduced, thus realizing the sustainable use of marine resources. Bisphenol A (BPA) is an additive to plastic products. Trace amounts of BPA can cause brain, cardiovascular, and reproductive system dysfunction and are highly associated with a variety of cancers. Light-driven MXene-based microrobots remove BPA from water, as reported by Dekanovsky et al. MXene was used as a scaffold grafted with bismuth nanoparticles that acted as photocatalysts. The microrobots are embedded with Fe_2_O_3_ as a second driving engine. When exposed to light, reactive oxygen species are generated to decompose BPA, and the resulting bubbles generate propulsion for the light drive. The microrobots can remove BPA efficiently with the optical-magnetic coupling drive. This research may pave the way for the degradation of new pollutants such as BPA (Figure 9D) [35].

The application of microrobots in the field of environmental protection is focused on the removal of pollutants from water bodies. This technology is in a phase of rapid development. Several research teams are working on the development of microrobots to remove harmful chemicals from water bodies. They can precisely locate and identify pollutants in water bodies and perform cleanup tasks. At the same time, the microrobots can be remotely maneuvered through various fields, enabling them to move around the water body and complete cleaning operations. Although these technologies are still in the research and development stage, they have already shown great potential. In the future, with the continuous innovation and improvement of the technology, it is believed that microrobots will play an increasingly important role in the field of water pollution control.

## 5. Summary, Current Challenges, and Future Work

In this paper, we systematically explore three key aspects of microrobots powered by multi-physics fields: the principles of material structure and response, forms of motion, and applications in medicine and environmental protection. Microrobots powered by multi-physics fields have many advantages compared with microrobots powered by single-physics fields. First, microrobots powered by multi-physics fields can be controlled and navigated in a variety of ways, making them more flexible in complex environments. Secondly, using a variety of physical fields to drive microrobots can improve the robustness of the system, making it more adaptable to external disturbances and changes. Finally, microrobots powered by multi-physics fields can use multiple physical fields simultaneously to achieve different functions, such as using magnetic fields to drive and release drugs in response to changes in pH. Therefore, microrobots powered by multi-physics fields have obvious advantages in flexibility, robustness, and versatility compared with single-field driven microrobots.

In the future, microrobots powered by multi-physics fields as a multifunctional and efficient micro–nano technology show great application potential and development prospects:

1. The continuous progress of new materials will provide a strong driving force for the miniaturization and efficiency of microrobots. For example, the application of nanomaterials allows microrobots to achieve smaller sizes and lighter designs while increasing their strength and flexibility. This will make microrobots more refined in size while improving their performance and efficiency;
2. The manufacturing technology of microrobots is also improving. The development of 3D printing technology has made the manufacturing process of microrobots more flexible and efficient, capable of achieving more complex structures and fine designs, thus further promoting the development of microrobots;
3. Microrobots need to have greater perception and decision-making capabilities in order to respond flexibly in a changing environment. This will require microrobots to be able to pick up information about their surroundings and respond to it. This ability is important in the medical field. For example, targeted drug delivery to cancer cells can dramatically improve treatment effectiveness;
4. Microrobots will become important tools in the field of biomedicine. For example, they can be used for drug delivery to precisely deliver drugs to diseased sites, thereby minimizing damage to healthy tissue. In addition, microrobots can also be used in minimally invasive surgery to enter the human body through tiny incisions for diagnosis and treatment, reducing patient pain and recovery time. As the technology develops, they will open up new possibilities for medical treatments;
5. The application of microrobots will make important contributions to the cause of environmental protection. They can carry out environmental remediation work, such as cleaning sediment at the bottom and collecting floating marine debris. This will effectively improve the quality of the environment.

In short, the future of microrobots powered by multi-physics fields will make breakthroughs in various fields and bring great changes to human life and work. Through continuous research and innovation, microrobots are expected to become an important tool to solve many practical problems and make positive contributions to the development of human society.

## Data Availability

No data were generated or used during the study.
